# A CASE REPORT OF UPPER EXTREMITY DVT PRESENTING AS METASTATIC LYMPHOMA

**DOI:** 10.51894/001c.123023

**Published:** 2024-08-30

**Authors:** Gretchen Yormick

**Affiliations:** 1 College of Osteopathic Medicine Michigan State University

## 55

### INTRODUCTION

Upper extremity DVT is defined as clot formation in veins such as the internal jugular and brachial veins. The etiology is not well understood but can be primary (hypercoagulable states) or secondary. Classic presenting symptoms include arm and facial edema, shoulder or neck discomfort, and limb heaviness. Although less common than lower extremity DVT, it is important to recognize and promptly treat to avoid complications such as pulmonary embolism.

### CASE DESCRIPTION

A 48-year-old female presented to the emergency department with chief complaints of shortness of breath, sore throat, left arm and neck swelling. Labs were remarkable for leukocytosis and elevated LDH. Physical examination showed left upper extremity tenderness and swelling. Initial CTA of the chest demonstrated prominent left neck and axillary lymph nodes, a small soft tissue lesion in the left breast and multiple focal areas of splenic hypodensities. Upper extremity doppler showed a DVT in the left internal jugular and subclavian veins. CT of the neck with contrast showed an infiltrating mass in the superior mediastinum extending into the left side of the neck with occlusion of the left subclavian and internal jugular veins. Given the patient’s initial imaging findings and clinical presentation, the concern for metastatic lymphoma was raised. Subsequent imaging including CT abdomen showed calcified splenic granulomas, ruling out splenic masses. CT venogram of the neck and repeat CTA of the chest revealed that the initial soft tissue abnormalities seen on CT were likely reactive, secondary to the thrombus. Breast ultrasound showed a benign left breast cyst and MRI of the chest showed no focal masses necessitating a biopsy. The patient started anticoagulation therapy, and on day 3 of admission, underwent successful mechanical thrombectomy and was discharged with outpatient follow-up.

### DISCUSSION

In reported cases of UEDVT, 38% of patients were found to have cancer. In this case, although initial imaging findings were concerning for a malignant process, repeat imaging was consistent with a reactive process secondary to an underlying thrombus. This case demonstrates the overlapping symptoms of upper extremity DVT and lymphoma and highlights the importance of follow-up imaging in establishing the final diagnosis.

